# CellExLink: End-to-end cell-type recognition and normalization in biomedical text

**DOI:** 10.1371/journal.pcbi.1014556

**Published:** 2026-07-24

**Authors:** Alimire Nabijiang, Leili Shahriyari

**Affiliations:** Department of Mathematics and Statistics, University of Massachusetts Amherst, Amherst, Massachusetts, United States of America; Teesside University, UNITED KINGDOM OF GREAT BRITAIN AND NORTHERN IRELAND

## Abstract

Cell types are described in biomedical literature using diverse names, abbreviations, and phenotype phrases, which complicates their recognition and normalization. We developed CellExLink, an end-to-end pipeline that identifies cell-type mentions and normalizes them to Cell Ontology (CL) identifiers. The recognizer was fine-tuned and evaluated on five heterogeneous biomedical corpora spanning full-length articles, article excerpts, figure captions, abstracts, and anatomical text passages. These resources include fine-grained phenotype-defined populations, heterogeneous cell populations, and abbreviated mentions. Across the five corpora, CellExLink achieved macro-average exact- and relaxed-span F1 scores of 0.766 and 0.855, respectively. For CL identifier normalization on gold-standard mention spans, F1 scores ranged from 0.690 to 0.874. In strict end-to-end evaluation, which required both an exact mention span and the correct CL identifier, F1 scores ranged from 0.552 on a figure-caption corpus to 0.725 on a corpus of full-text article excerpts. CellExLink outperformed the evaluated off-the-shelf systems in cell mention recognition, CL identifier normalization, and end-to-end extraction. By converting unannotated biomedical text into cell-type spans linked to standardized CL identifiers, CellExLink provides a practical foundation for downstream applications, including literature curation, relation extraction, and knowledge graph construction.

## Author summary

Cell types are central to biomedical research, but biomedical papers often use different names, abbreviations, and synonyms for the same cell type. This variation makes it difficult for automated processes to collect and compare cell-type information across papers. Reliable automated extraction is important because literature mining requires consistent cell-type identification before evidence from different studies can be searched, integrated, or reused. Existing off-the-shelf biomedical text-mining tools provide useful functionality, but their ability to support cell-type extraction remains limited and inconsistent. To address this gap, we developed CellExLink, a pipeline that finds cell-type entities in biomedical text and links them to standard Cell Ontology identifiers. We evaluated the pipeline on several biomedical corpora and compared it with existing tools that support cell-type extraction. Across these evaluations, CellExLink showed clear accuracy gains in both detecting cell-type entities and assigning correct standard identifiers. Together, these gains make CellExLink useful for extracting more reliable standardized cell-type information from large collections of papers, supporting literature curation, relation extraction, knowledge graph construction, and studies of cell-type-specific roles in diseases, drug responses, and biological pathways.

## Introduction

Cells are the fundamental units of biology, and many other biological entities are interpreted in relation to specific cell types [1,2]. The importance of cell types is particularly evident in biological processes and disease states governed by interactions among diverse cellular and molecular components [3–7]. The computational task of identifying cell types in published literature is important for advancing biomedical research and healthcare-related computational modeling. The need for accurate cell-type extraction is illustrated by the tumor microenvironment, where interactions among diverse cell types, including tumor cells, immune cells, and stromal cells, influence disease progression and treatment response [8–11]. These interactions can be modeled using quantitative systems pharmacology (QSP) approaches to predict tumor progression and treatment response [12–16]. The predictive value of such models depends on the accuracy and completeness of the underlying tumor microenvironment interaction network. Because these networks are often constructed from biomedical literature, reliable cell-type extraction is an important first step toward building more accurate interaction networks and supporting downstream computational modeling [12,14,17].

The task of extracting cell-type entities from literature has two linked parts: (i) named entity recognition (NER), which identifies entities in text, such as cell types or cell populations, and (ii) named entity normalization (NEN), also called entity linking, which maps each entity to a standard identifier. The entity-normalization step is needed because the same concept can appear in many forms in text. For example, a cell type may be represented by alternative names or abbreviations, such as “granulosa cell” or “GC,” and “endothelial cell” or “endotheliocyte.” Normalization maps these forms to the same Cell Ontology (CL) identifier [18], which improves consistency across documents and supports downstream analysis.

Current off-the-shelf biomedical text-mining tools offer useful capabilities, but their support for cell-type recognition and normalization remains uneven. PubTator3 [19] recognizes genes/proteins, chemicals, diseases, species, variants, and cell lines, but not cell types. HunFlair2 [20] supports cell lines, chemicals, diseases, genes, and species but does not target cell types. GNorm2 [21], a strong system for gene recognition and normalization, is limited to genes. VANER2 [22] expands biomedical NER coverage, but it is a recognition system and does not link entities to ontology identifiers. Although the more comprehensive BERN2 system [23] includes both cell-type recognition and normalization, its normalization strategy relies on rule-based methods, which have limited coverage. The scispaCy toolkit [24] provides biomedical NER models with cell-related labels. However, its built-in linker does not support the Cell Ontology. As a result, an external integration such as PyOBO [25] is needed for CL linking. Here, we present an informatics tool that addresses an unmet need for practical, end-to-end support for both cell-type recognition and Cell Ontology normalization.

Furthermore, the advances in biomedical research indicate the need for new biomedical entity recognition tools. For instance, recent advances in single-cell technologies have changed how authors describe cell phenotypes [2,26,27]. Earlier corpora with cell-related annotations, including AnatEM [28], CRAFT [29], JNLPBA [30], and BioID [31], are still useful. However, recent work by Rotenberg et al. [27] suggests that these corpora do not capture the full range of fine-grained phenotypes reported in newer single-cell-focused papers. In that study, CellLink, built from literature published between 2019 and 2024, reported more than 22,000 annotated entities, 9,804 unique entities, and 1,251 unique Cell Ontology identifiers, covering nearly half of the current Cell Ontology. By comparison, BioID and CRAFT together contain 378 unique Cell Ontology identifiers. This shift suggests that systems trained only on earlier resources may not be able to identify many cell types found in recent papers and may not provide broad enough coverage of Cell Ontology identifiers.

Model selection is an important consideration when developing an informatics tool for cell-type recognition and normalization. Recent advances in deep learning have made pretrained transformer models central to biomedical natural language processing (NLP). These models include encoder-based biomedical language models, such as PubMedBERT and Bioformer, as well as generative large language models (LLMs) [32–37]. However, recent studies suggest that generative LLMs do not consistently outperform strong fine-tuned encoder-based biomedical models on information-extraction tasks, although they can be competitive on reasoning-oriented tasks such as question answering [22,38,39]. In addition, LLMs are generally more computationally expensive at inference [22]. Furthermore, NER and NEN are often the first stages of larger biomedical text-mining pipelines and are applied repeatedly across large corpora. Therefore, their efficiency can affect the runtime of the overall pipeline [33]. For this reason, cell-type extraction systems need to consider computational efficiency alongside performance, motivating the use of fine-tuned encoder-based biomedical transformers.

Guided by these considerations, we developed CellExLink [40], a cell-type-specific end-to-end pipeline that converts unannotated biomedical text into cell-type mention spans linked to Cell Ontology identifiers. The main contribution of CellExLink is a practical and reusable workflow for cell-type extraction that combines recognition and normalization in a single system while tailoring both stages to the Cell Ontology task. For the recognition stage, we compared PubMedBERT [32] and Bioformer16L [33] under four training-data composition strategies across five heterogeneous corpora and selected jointly fine-tuned Bioformer16L as the final recognizer based on its balance of accuracy, model size, and inference latency. For the normalization stage, we evaluated multiple dense-retrieval backbones and developed a Cell Ontology–aware retrieval and reranking framework rather than relying on an off-the-shelf SapBERT model alone. Specifically, we adapted SapBERT [41] to Cell Ontology terminology and augmented dense retrieval with ontology labels and synonyms, abbreviation-aware matching, and candidate reranking. This combination enabled more robust linking of extracted cell mentions to standardized Cell Ontology concepts. Because the learned backbone is a major computational component of each stage, backbone selection was guided by both predictive performance and computational efficiency. CellExLink connects these stages in a sequential workflow that processes unannotated text without manual transfer of intermediate outputs. We evaluated CellExLink at three complementary levels: cell-type recognition, Cell Ontology normalization on gold-standard mention spans, and strict end-to-end extraction. This design allowed recognition performance, normalization performance, and full-pipeline performance to be assessed separately.

## Methods

### Overview

**Fig 1:**
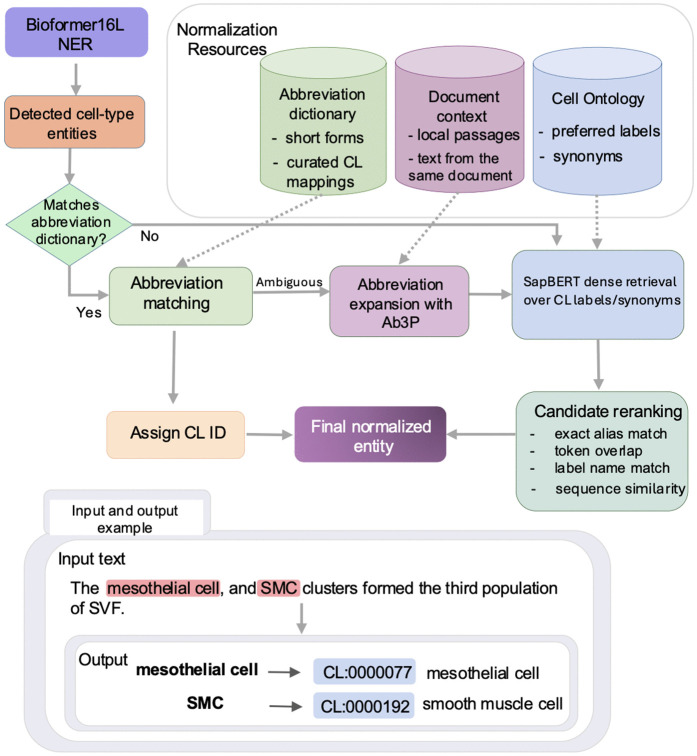
Overview of CellExLink. Fine-tuned Bioformer16L [33] detects cell-type mention spans, which are passed directly to the normalization component. Fine-tuned SapBERT [41] is the main normalization backbone: it embeds each detected mention and retrieves candidate Cell Ontology concepts by vector similarity over CL preferred labels and synonyms. For detected mentions that match the abbreviation dictionary, CellExLink assigns an identifier directly only when the dictionary contains a unique CL mapping; dictionary-ambiguous abbreviations are expanded with Ab3P [42] using document-local text when available. CellExLink then reranks candidate concepts using the dense-retrieval score and lexical features and returns the top-ranked CL identifier.

CellExLink is an end-to-end pipeline composed of two sequential components: (i) a cell-type NER component and (ii) a Cell Ontology normalization component. The recognition component identifies cell-type spans in the input text, and the normalization component maps each detected mention to a Cell Ontology identifier. In the end-to-end workflow, these components are executed consecutively without manual transfer of their outputs (Fig 1).

### Datasets and evaluation design

We used five annotated biomedical corpora: CellLink [27], BioID [31], CRAFT [29], AnatEM [28], and JNLPBA [30] (Table 1). CellLink contains annotations categorized as cell phenotype, heterogeneous cell population, or cell_vague. We retained the cell-phenotype and heterogeneous-cell-population annotations and excluded cell_vague annotations because they describe nonspecific cell populations that cannot be resolved to precise CL concepts. Training and evaluation partitions remained separate for each corpus, and only designated training records were used for parameter updates. For CellLink, the validation set served as the evaluation partition because ground-truth labels for the test set were not available. For the other four corpora, we used the designated test sets.

**Table 1:** Datasets used in this study. The datasets include CellLink [27], BioID [31], JNLPBA [30], CRAFT [29], and AnatEM [28]. Normalization and end-to-end evaluation were limited to datasets with CL identifiers. For CellLink, the validation set was used instead of the test set.

Dataset	Text type	Origin / release	Corpus statistics (train / test)			CL Identifier
			Annotations	Unique entities	Documents	
CellLink	Full-text article excerpts	Biomedical articles, 2019–2024	11,140 / 3,591	5,410 / 2,203	1,502 / 501	Yes
BioID	Figure panel captions	SourceData / BioCreative VI Bio-ID, 2017	3,522 / 1,094	514 / 183	10,889 / 2,808	Yes
CRAFT	Full-length journal articles	PubMed Central, 2012 release	3,462 / 1,749	661 / 393	60 / 30	Yes
AnatEM	Abstracts and text extracts	Extended anatomical entity corpus, 2014	1,787 / 1,031	766 / 530	603 / 202	No
JNLPBA	MEDLINE abstracts	GENIA v3.02 shared-task corpus, 2004	6,710 / 1,909	2,141 / 940	1,999 / 403	No

To characterize corpus-level coverage by the Cell Ontology, we also analyzed unique annotated mention surface forms from the CL-annotated corpora listed in Table 1 (CellLink, BioID, and CRAFT; S1 Fig). Overall, 97.7% of CellLink, 96.7% of BioID, and 99.4% of CRAFT mention forms were associated with existing Cell Ontology concepts after combining exact lexical matching to CL preferred labels or synonyms with resolution of corpus-provided CL identifiers. The remaining unresolved no-exact lexical-match forms are candidates for expert review as possible missing synonyms or ontology gaps.

We evaluated CellExLink at three levels. First, we evaluated cell-type recognition on all five corpora using exact-span and relaxed-span matching. Exact-span matching required identical predicted and gold mention boundaries, whereas relaxed-span matching required token-level overlap. To avoid giving credit to overly broad partial predictions, we excluded generic predictions such as “cell,” “cells,” and “cellular” from relaxed matches. Second, we evaluated CL normalization on gold-standard mention spans, which isolates normalization performance from recognition errors. Third, we evaluated strict end-to-end extraction, which required both an exact mention span and the correct CL identifier. This evaluation captures recognition errors, normalization errors, and error propagation between the two stages.

We evaluated CL normalization and strict end-to-end extraction only on CellLink, BioID, and CRAFT because these corpora provide CL identifiers. We included AnatEM and JNLPBA in the recognition evaluation but excluded from CL normalization and end-to-end evaluation because they do not provide CL identifiers.

We compared CellExLink with off-the-shelf systems according to the functions supported by each system (Table 2). We evaluated BERN2 and scispaCy in all supported settings. However, we included VANER2 only in the recognition evaluation because it does not provide entity normalization.

**Table 2:** Functionality of the systems evaluated in this study. Comparisons were restricted to the functions supported by each system. VANER2 was included only in the NER-only comparison because it does not provide entity normalization.

System	NER	NEN	Backbone (params)	Year
BERN2	✓	✓	BioLM (~365M)	2022
VANER2	✓	×	Llama 3.1 Instruct (~8B)	2026
scispaCy	✓	✓	spaCy pipeline	2019/2025(updated)
**CellExLink**	**✓**	**✓**	BioFormer16L + SapBERT (~150M)	2026

### Cell-type recognition

To develop an accurate and computationally efficient NER component, we evaluated two biomedical transformer encoders as candidate recognition backbones: PubMedBERT [32], a widely used biomedical language model that served as a strong high-capacity baseline, and Bioformer16L [33], a substantially smaller biomedical encoder designed to provide efficient inference while retaining competitive performance. We selected these models to assess the trade-off between recognition accuracy and computational efficiency for large-scale literature processing. We evaluated both backbones using the same flat NER formulation, training data, and token-classification architecture.

For each backbone, we formulated NER as a flat beginning-inside-outside (BIO) sequence-labeling task. After subword tokenization, a token-classification head assigned a beginning-of-entity (B), inside-entity (I), or outside (O) label to each subword token, excluding special tokens. We then used the tokenizer offsets to convert the predicted labels into character-level mention spans. Because each labeled token receives only one BIO label, the model produces non-overlapping spans and cannot represent nested or overlapping mentions at the same text location; only the predicted spans are passed to the normalization component.

To leverage the new CellLink dataset [27], we explored fine-tuning under different data compositions. We treated CellLink as a separate data source because it represents more recent, phenotype-rich literature, and we merged CRAFT, BioID, AnatEM, and JNLPBA as the earlier-corpus source. We compared four fine-tuning strategies: (i) fine-tuning on CellLink alone; (ii) fine-tuning on the concatenated training records from the four earlier corpora; (iii) fine-tuning first on the concatenated earlier-corpus training records and then further fine-tuning on CellLink; and (iv) joint fine-tuning on the concatenated training records from all five corpora. We did not manually alter retained text, adjust entity boundaries, or resample corpora.

For model selection, we considered recognition performance, model size, and inference latency. We computed micro-F1 separately for each corpus under exact-span and relaxed-span matching, and then macro-averaged the five corpus-level F1 scores. We selected Bioformer16L jointly fine-tuned on the combined training portions of all five corpora as the final recognizer. The CellExLink version 1.0.0 release includes code documenting the preprocessing and fine-tuning workflow [40].

### Cell-type normalization

We normalized each recognized mention to a CL identifier using CellExLink’s normalization stage. A CL class has a unique identifier, one preferred label, and zero or more synonyms. We refer to each CL class as a concept and to its preferred label and synonyms collectively as its aliases.

For the normalization component, we used three resources. First, we constructed a CL label-and-synonym inventory by retrieving the preferred-label and synonym fields for classes in Cell Ontology release v2025-12–17 [43] through the BioPortal representational state transfer application programming interface (REST API) [44]. These aliases served as candidate strings for dense retrieval. Second, we constructed a cell-type abbreviation dictionary from abbreviation-like mentions and their CL mappings in the fine-tuning data. Third, when abbreviation resolution was needed, we used the complete text of the input document to recover explicit abbreviation–expansion pairs with Ab3P [42].

The final normalization stage uses fine-tuned SapBERT [41] as the dense-retrieval backbone; the backbone-selection experiments are reported in results and discussion. SapBERT uses self-alignment pretraining to place biomedical names associated with the same Unified Medical Language System (UMLS) concept near one another in the embedding space. In CellExLink, SapBERT serves as the learned dense-retrieval backbone. We adapted the pretrained SapBERT model using supervised contrastive learning on concept-labeled names assembled from CL preferred labels, CL synonyms, and CellLink training mentions with exact CL mappings. During fine-tuning, we treated names associated with the same CL identifier as positive examples.

At inference time, CellExLink first applies abbreviation handling to spans already identified as cell-type mentions. We considered an abbreviation key unambiguous only when every entry for that key in the cell-type abbreviation dictionary mapped to the same CL identifier. Such mentions are normalized directly to that identifier. This uniqueness criterion is internal to the cell-type dictionary and does not imply that the abbreviation has no non-cell meaning elsewhere in biomedical text.

For a dictionary key associated with multiple CL identifiers, we utilized Ab3P to search passages from the same document for a matching abbreviation–expansion pair. When an expansion is recovered, it replaces the abbreviation as the dense-retrieval query. When the key is absent from the dictionary or an ambiguous key has no recoverable expansion, it uses the original mention text. It therefore uses document context specifically to recover explicit abbreviation expansions. It independently normalizes each recognized span. For example, if “mesothelial cell of intestine” and “mesothelial cell” occur as separate recognized mentions, each is queried using its own span text, and string containment alone does not create an abbreviation or replacement relation between them. If two candidate mentions overlap at the same text location, the flat BIO recognizer outputs only one non-overlapping span, and only that predicted span is passed to normalization.

For every mention not assigned directly through the abbreviation dictionary, fine-tuned SapBERT independently encodes the query and all CL aliases. Cosine similarity produces alias-level matches, which are grouped by CL identifier. The highest alias-level cosine similarity is retained as the concept-level dense-retrieval score. We used the maximum because aliases are alternative names for the same concept: a mention may closely match one valid alias while differing from the others. Summing scores would favor concepts with more aliases, whereas averaging could dilute a strong match to one valid alias. Candidate concepts are then reranked using the dense-retrieval score and lexical features, including exact synonym match, token overlap, preferred-label overlap, and sequence similarity. The top-ranked concept are returned as the final CL identifier.

## Results and discussion

To evaluate CellExLink, we first compared the recognition component and its backbone fine-tuning strategies, then assessed the dense-retrieval backbones used for normalization and the complete normalization procedure, and finally reported full-pipeline performance and illustrative examples.

### Cell-type recognition

CellExLink outperformed all evaluated off-the-shelf systems under both exact-span and relaxed-span matching on every corpus (Table 3). BERN2 was the strongest off-the-shelf baseline under exact-span matching. VANER2 was more competitive under relaxed-span matching than under exact-span matching, indicating that many of its errors involved span boundaries rather than complete misses.

**Table 3:** NER-only cell-type recognition performance. Performance was measured on five corpora using micro-F1 under exact-span (E) and relaxed-span (R) matching. Macro average denotes the average of per-corpus micro-F1 across the five corpora.

System	CellLink		CRAFT		BioID		AnatEM		JNLPBA		Macro avg.	
	E	R	E	R	E	R	E	R	E	R	E	R
scispaCy	0.308	0.488	0.375	0.462	0.136	0.181	0.248	0.334	0.222	0.382	0.258	0.369
BERN2	0.684	0.847	0.406	0.592	0.244	0.413	0.567	0.731	0.663	0.778	0.513	0.672
VANER2	0.430	0.837	0.180	0.566	0.315	0.452	0.302	0.813	0.318	0.741	0.309	0.682
**CellExLink**	**0.860**	**0.941**	**0.740**	**0.840**	**0.745**	**0.800**	**0.764**	**0.864**	**0.723**	**0.832**	**0.766**	**0.855**

**Fig 2:**
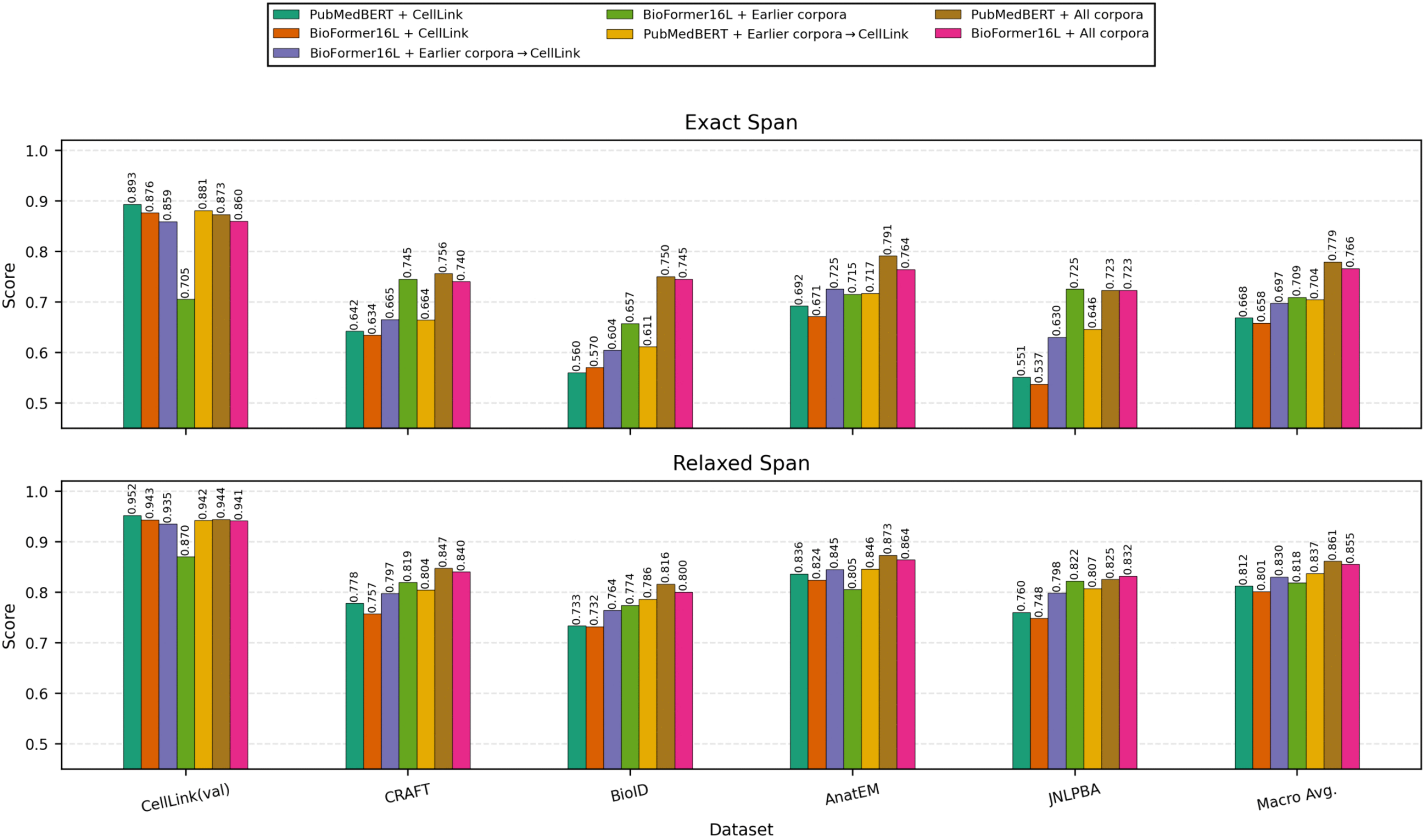
Effect of fine-tuning data and encoder choice on cell-type recognition. The upper panel shows exact-span micro-F1, the lower panel shows relaxed-span micro-F1, and the rightmost column in each panel shows the macro average across corpora.

Fine-tuning performance depended strongly on data composition (Fig 2 and S1 Table). Training on CellLink alone produced strong CellLink performance but transferred less effectively to CRAFT, BioID, AnatEM, and JNLPBA. Training on the four earlier corpora generally improved those corpora while reducing performance on CellLink. Sequential fine-tuning on the earlier corpora and then CellLink recovered CellLink performance but did not provide the best overall balance. For both PubMedBERT and Bioformer16L, joint fine-tuning on all five corpora produced the highest macro-average performance.

Among the jointly fine-tuned models, PubMedBERT achieved exact-span and relaxed-span macro-F1 scores of 0.779 and 0.861, compared with 0.766 and 0.855 for Bioformer16L. Bioformer16L, however, used 42 million rather than 100 million parameters and required 19.602 rather than 46.628 ms per abstract (Table 4). We therefore selected jointly fine-tuned Bioformer16L as the final recognizer because its small reduction in F1 was offset by substantially lower model size and latency.

**Table 4:** Model size and inference latency of candidate backbones.

Task	Model	Size (M)	Inference latency
NER	Bioformer16L	42	19.602 ms/abstract
NER	PubMedBERT	100	46.628 ms/abstract
NEN	Bioformer16L	42	5.601 ms/entity
NEN	SapBERT	100	9.604 ms/entity
NEN	BioLORD	110	10.561 ms/entity
NEN	MedCPT-Query	100	9.402 ms/entity

Measurements were taken on an NVIDIA A16 GPU. Model size is reported in millions of parameters (M). Inference latency is reported in milliseconds per abstract for NER and milliseconds per entity for NEN. The JNLPBA dataset was used.

### Cell-type normalization on gold-standard spans

On gold-standard spans, CellExLink outperformed BERN2 and scispaCy in every evaluation setting (Table 5). Because all systems received the same reference spans, these results isolate CL identifier assignment from recognition performance. The highest CellExLink F1 was 0.874 on the CellLink exact-only subset, and the lowest was 0.690 on BioID figure captions.

**Table 5:** Cell-type normalization performance on gold-standard entity spans, reported as F1.

Dataset	BERN2	scispaCy	CellExLink
**CellLink (exact matches only)**			
Cell phenotype	0.342	0.577	**0.872**
Heterogeneous cell population	0.356	0.544	**0.939**
Overall	0.342	0.576	**0.874**
**CellLink (all IDs)**			
Cell phenotype	0.285	0.515	**0.786**
Heterogeneous cell population	0.174	0.363	**0.601**
Overall	0.278	0.506	**0.775**
CRAFT	0.331	0.490	**0.818**
BioID	0.220	0.457	**0.690**

Table 6 separates the evaluation of zero-shot dense-retrieval backbones, task-adapted dense retrieval, and the complete CellExLink normalization procedure. We selected the normalization backbone by considering F1 on gold-standard mention spans, model size, inference latency, and suitability for synonym-based concept retrieval. No zero-shot encoder performed best in every setting: SapBERT performed best on CellLink exact-only, MedCPT-Query [45] on CRAFT, and BioLORD [46] on the CellLink all-identifiers setting and BioID. SapBERT had latency comparable to that of MedCPT-Query and lower than that of BioLORD (Table 4), and its synonym-alignment pretraining directly matched the CL retrieval task; we therefore selected it for task-specific adaptation.

**Table 6:** Dense-retrieval backbone selection and complete CellExLink normalization performance. Results are F1 on gold-standard mention spans.

Configuration	CellLink		CRAFT	BioID
	Exact-only	All-ID		
*Zero-shot dense-retrieval backbones*				
SapBERT	0.787	0.662	0.750	0.534
MedCPT-Query	0.750	0.653	0.755	0.571
BioLORD	0.752	0.664	0.713	0.647
*Task-adapted dense-retrieval backbones*				
Bioformer16L (fine-tuned)	0.783	0.692	0.743	0.620
SapBERT (fine-tuned)	0.825	0.728	0.802	0.645
*Complete normalization procedure*				
**CellExLink**	**0.874**	**0.775**	**0.818**	**0.690**

Fine-tuned Bioformer16L was smaller and faster than fine-tuned SapBERT, but fine-tuned SapBERT achieved higher F1 across all normalization evaluations. Fine-tuning improved SapBERT over its zero-shot version in every setting, and adding abbreviation handling and candidate reranking produced further gains, yielding the complete CellExLink normalization procedure.

### Strict end-to-end extraction

Strict end-to-end evaluation requires both an exact span and the correct CL identifier (Table 7). CellExLink achieved F1 scores of 0.725 on CellLink, 0.667 on CRAFT, and 0.552 on BioID, and substantially outperformed both baselines across all three evaluation datasets. The gap between gold-span normalization and end-to-end extraction indicates that recognition errors remained an important source of performance loss. Much of this loss came from missed mentions and span-boundary errors, which reduced recall. Overall, CellExLink still outperformed both baseline tools by a large margin.

**Table 7:** Strict end-to-end cell-type extraction performance. A prediction was counted as correct only when both the mention span and the Cell Ontology identifier were correct. Scores are reported as linked precision (P), recall (R), and F1.

System	CellLink			CRAFT			BioID		
	P	R	F1	P	R	F1	P	R	F1
scispaCy	0.231	0.228	0.229	0.168	0.183	0.175	0.064	0.166	0.093
BERN2	0.728	0.103	0.181	0.865	0.070	0.129	0.629	0.042	0.079
**CellExLink**	**0.733**	**0.716**	**0.725**	**0.788**	**0.579**	**0.667**	**0.622**	**0.496**	**0.552**

### Qualitative comparison of illustrative examples

S2 Fig presents illustrative examples comparing CellExLink with scispaCy and BERN2. The examples show both strengths and limitations of CellExLink. In examples 1–3, CellExLink recovered coordinated or fine-grained mentions that one or both baselines missed, mapped to broader concepts, or left unlinked. The remaining examples highlight challenges in short, caption-style passages, where surrounding context is limited and abbreviations are frequent. For example, in example 5, CellExLink consistently linked “mesothelial cell”/“mesothelial cells” and “SMC”/“SMCs,” but linked “EC”/“ECs” to incorrect Cell Ontology identifiers. Other examples show boundary-related errors, including left-boundary overextension or partial recovery of a longer gold mention. These examples suggest that abbreviation ambiguity and exact boundary recovery remain important sources of error in context-limited text; they are intended to illustrate failure modes rather than estimate their frequency.

## Conclusion

In this work, we presented CellExLink, an end-to-end pipeline for literature-based cell-type extraction that combines cell-type recognition with Cell Ontology normalization in a single sequential workflow. Across multiple evaluation corpora, CellExLink improved cell-type recognition, normalization, and strict end-to-end extraction relative to currently available off-the-shelf systems that support cell-type extraction.

This work has limitations that can be addressed in future studies. For example, the current annotation setup used flat, non-overlapping entity annotations, which may not fully represent overlapping or nested entities. Therefore, some entities may have been simplified or not fully captured. Short figure captions and abbreviation-heavy text also remained challenging because they provide limited context for both span detection and normalization. In addition, although every evaluation document was excluded from parameter training, evaluation on a fully external corpus would provide a stronger independent test of generalization. Future work can improve cell-type recognition and normalization by supporting richer annotation structures, enhanced abbreviation handling, stronger boundary detection, and evaluation on external corpora.

## Supporting information

S1 Fig Corpus mention coverage by existing Cell Ontology concepts in CL-annotated corpora. (PDF)

S1 Table Recognition performance of the candidate encoders under the four fine-tuning strategies. Exact-span and relaxed-span F1 scores are reported for PubMedBERT and Bioformer16L under each evaluated training-data configuration.
(PDF)

S2 Fig Qualitative comparison of illustrative outputs from CellExLink, scispaCy, and BERN2. (PDF)
