# Supplementary material for: CellExLink: End-to-end cell-type recognition and normalization in biomedical text
Source: PLoS Comput Biol. 2026 Jul 24;22(7):e1014556. doi: 10.1371/journal.pcbi.1014556 (PMC13436769; doi:10.1371/journal.pcbi.1014556)
Supplement: Supplementary file 1 [file pcbi.1014556.s001.pdf]

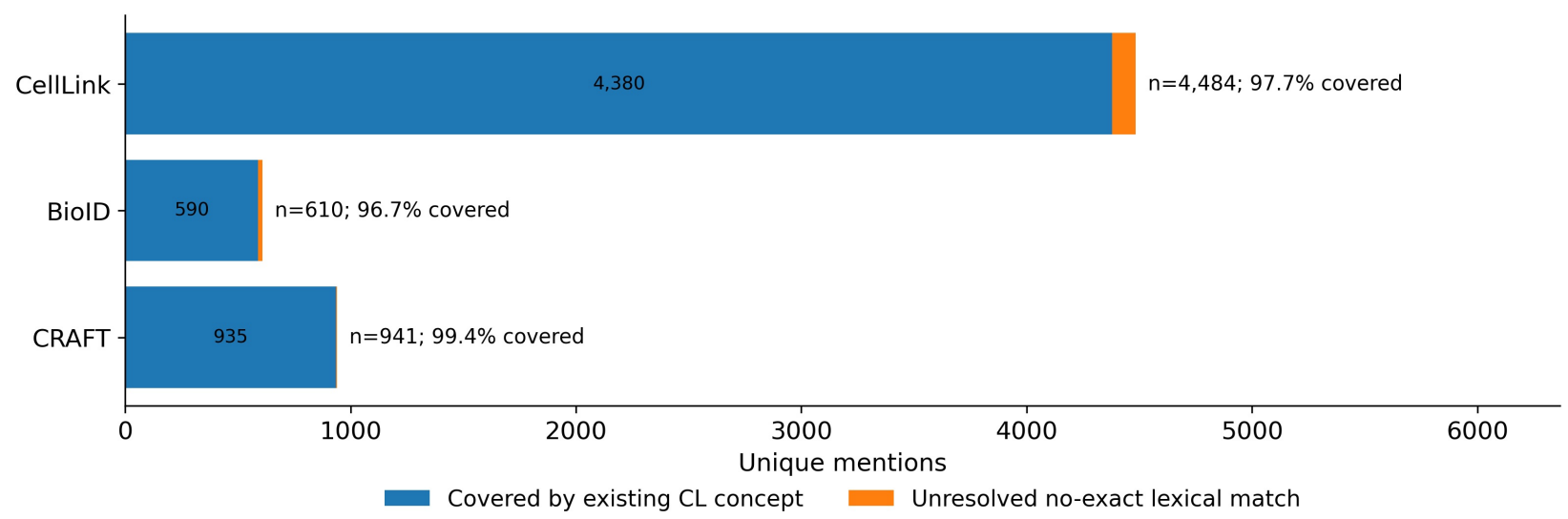

S1 Fig. Corpus mention coverage by existing Cell Ontology concepts in CL-annotated corpora. Bars show the number of unique annotated mention surface forms in the CL-annotated corpora from Table 1 that were covered by an existing Cell Ontology concept, compared with the smaller subset of unresolved no-exact lexical matches. A mention form was considered covered if it exactly matched a CL preferred label or synonym after conservative string standardization, including lowercasing, whitespace trimming, and simple plural handling, or if it had no exact lexical match but its corpus-provided CL identifier resolved to a CL concept. The analysis shows that 4,380 of 4,484 CellLink mention forms (97.7%), 590 of 610 BioID mention forms (96.7%), and 935 of 941 CRAFT mention forms (99.4%) were covered by CL concepts. The unresolved no-exact lexical-match category represents the subset most relevant to potential missing synonyms or ontology gaps for future expert review. AnatEM and JNLPBA are not shown because they do not provide CL identifiers. For CellLink, annotations labeled cell_vague were excluded because they describe nonspecific cell populations and do not provide CL identifiers.
