# Supplementary material for: CellExLink: End-to-end cell-type recognition and normalization in biomedical text
Source: PLoS Comput Biol. 2026 Jul 24;22(7):e1014556. doi: 10.1371/journal.pcbi.1014556 (PMC13436769; doi:10.1371/journal.pcbi.1014556)
Supplement: Supplementary file 2 [file pcbi.1014556.s002.pdf]

S1 Table. Recognition performance of the candidate encoders under the four fine-tuning strategies. Exact-span and relaxed-span F1 scores are reported for PubMedBERT and Bioformer16L under each evaluated training-data configuration.

Encoder	Fine-tuning strategy	CellLink		CRAFT		BioID		AnatEM		JNLPBA		Macro avg.	
		**E**	**R**	**E**	**R**	**E**	**R**	**E**	**R**	**E**	**R**	**E**	**R**
PubMedBERT	CellLink only	0.893	0.952	0.642	0.778	0.560	0.733	0.692	0.836	0.551	0.760	0.668	0.812
Bioformer16L	CellLink only	0.876	0.943	0.634	0.757	0.570	0.732	0.671	0.824	0.537	0.748	0.658	0.801
PubMedBERT	Earlier corpora only	0.732	0.881	0.754	0.822	0.663	0.794	0.722	0.809	0.732	0.830	0.721	0.827
Bioformer16L	Earlier corpora only	0.705	0.870	0.745	0.819	0.657	0.771	0.715	0.805	0.725	0.822	0.709	0.818
PubMedBERT	Earlier corpora → CellLink	0.881	0.942	0.664	0.804	0.611	0.786	0.717	0.846	0.646	0.807	0.704	0.837
Bioformer16L	Earlier corpora → CellLink	0.859	0.935	0.665	0.797	0.604	0.764	0.725	0.845	0.630	0.798	0.697	0.830
PubMedBERT	All corpora (joint)	0.873	0.944	0.756	0.847	0.750	0.816	0.791	0.873	0.723	0.825	0.779	0.861
Bioformer16L	All corpora (joint)†	0.860	0.941	0.740	0.840	0.745	0.800	0.764	0.864	0.723	0.832	0.766	0.855

Earlier corpora comprise CRAFT, BioID, AnatEM, and JNLPBA. The arrow denotes sequential fine-tuning, whereas “all corpora” denotes joint fine-tuning. †Final recognition model used for CellExLink.
