# Supplementary material for: CellExLink: End-to-end cell-type recognition and normalization in biomedical text
Source: PLoS Comput Biol. 2026 Jul 24;22(7):e1014556. doi: 10.1371/journal.pcbi.1014556 (PMC13436769; doi:10.1371/journal.pcbi.1014556)
Supplement: Supplementary file 3 [file pcbi.1014556.s003.pdf]

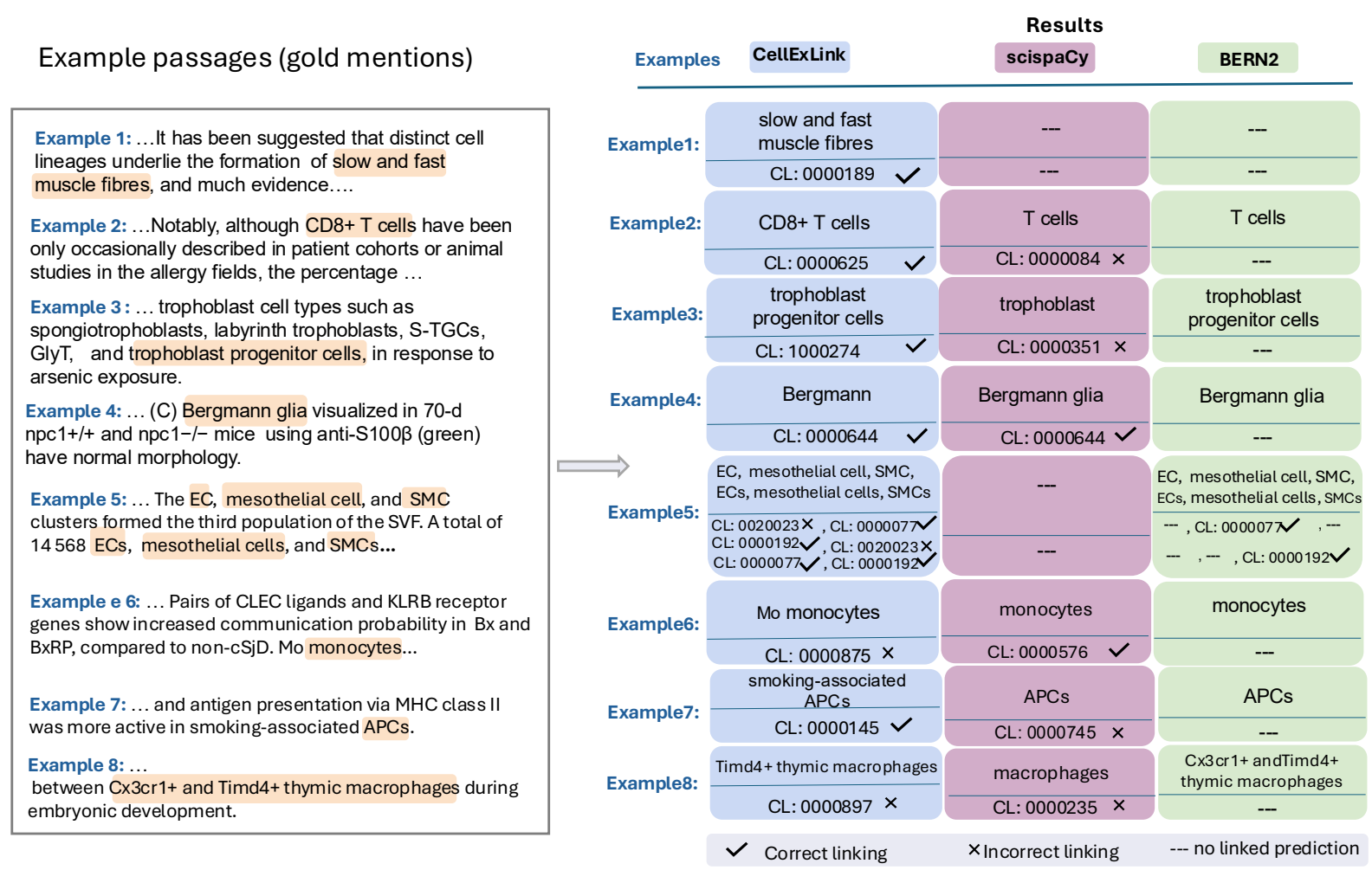

S2 Fig. Qualitative comparison of illustrative outputs from CellExLink, scispaCy, and BERN2. The left panel shows passages with gold-standard mentions highlighted. The right panel shows the mention spans and Cell Ontology identifiers returned by each system when available. Check marks indicate correct linking, crosses indicate incorrect linking, and dashes indicate no linked prediction.
